# Regulating life after death: how mechanical communication mediates the epithelial response to apoptosis

**DOI:** 10.1140/epje/s10189-022-00163-9

**Published:** 2022-01-25

**Authors:** Alexis Bonfim-Melo, Kinga Duszyc, Guillermo A. Gomez, Alpha S. Yap

**Affiliations:** 1 Division of Cell and Developmental Biology, Institute for Molecular Bioscience, The University of Queensland, St. Lucia, Brisbane, QLD 4072 Australia; 2 Centre for Cancer Biology, SA Pathology and University of South Australia, Adelaide, 5000 Australia; 3 Present Address: The University of Queensland Diamantina Institute, Faculty of Medicine, The University of Queensland, Brisbane, QLD 4102 Australia

## Abstract

**Abstract:**

It is increasingly evident that cells in tissues and organs can communicate with one another using mechanical forces. Such mechanical signalling can serve as a basis for the assembly of cellular communities. For this to occur, there must be local instabilities in tissue mechanics that are the source of the signals, and mechanisms for changes in mechanical force to be transmitted and detected within tissues. In this review, we discuss these principles using the example of cell death by apoptosis, when it occurs in epithelia. This elicits the phenomenon of apical extrusion, which can rapidly eliminate apoptotic cells by expelling them from the epithelium. Apoptotic extrusion requires that epithelial cells detect the presence of nearby apoptotic cells, something which can be elicited by the mechanotransduction of tensile instabilities caused by the apoptotic cell. We discuss the central role that adherens junctions can play in the transmission and detection of mechanical signals from apoptotic cells.

**Graphical abstract:**

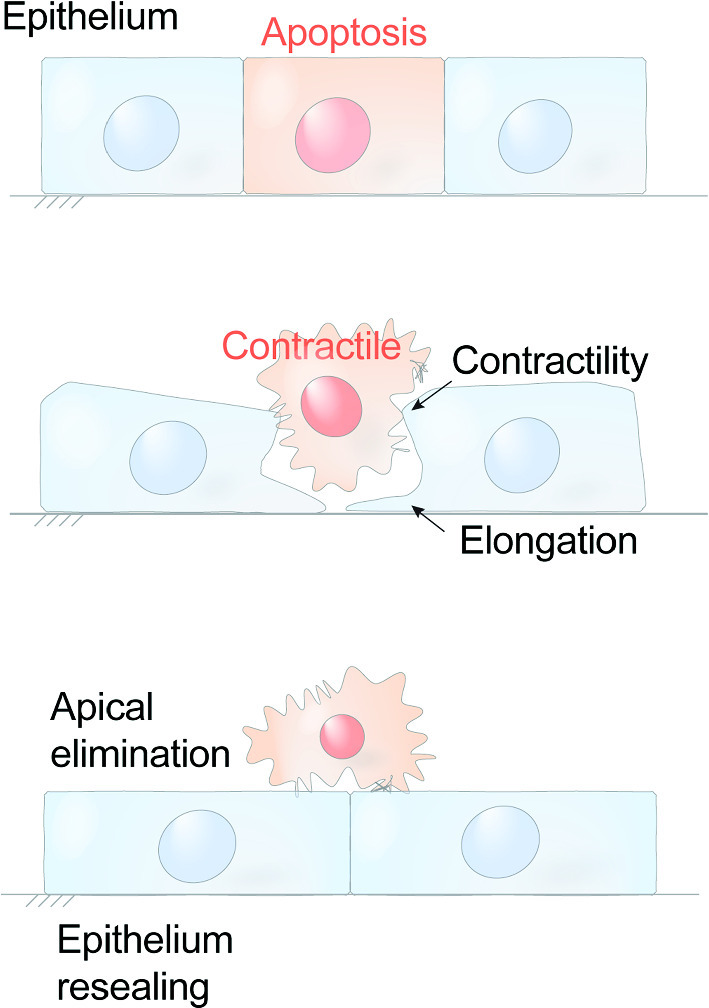

## Introduction

Multicellular organisms require mechanisms to coordinate the active, individual agency of their many constituent cells, linking them into coherent communities that share common functions or behaviours. Biological studies over the past two centuries have elucidated many processes responsible for such cell-to-cell coordination. These include the transmission of electrical signals by neurons [1]; long-range communication throughout the body mediated by hormones [2]; and shorter-range signals, such as nitric oxide and purine nucleotides, that are secreted by cells into their extracellular environment [3, 4]. All these diverse mechanisms share the hallmark of allowing cells to communicate with one another to generate communities. The scale of those communities may vary, from the local neighbourhoods that arise from short-range signalling to the whole-of-body coordination that is elicited by hormones.

It has recently become apparent that mechanical forces are also used as a mode of communication to create cellular communities. Specifically, mechanical forces generated by cells can be detected by other cells to alter their behaviour [5–7]. We can think of these communities as mechanobiomes, by analogy with the many “omes” (e.g. microbiome) that are used to denote biological communities. The notion of a mechanobiome implies that mechanical signalling influences the behaviour of multicellular populations. For this to occur, we need to identify the source and nature of the mechanical signal, the way it may be transmitted to other cells, and the mechanotransduction pathways that detect the signal and elicit the cellular response. To illustrate this in the present article, we discuss the example of how epithelia respond to apoptosis, a distinctive form of cell death [8, 9]. Here, an important role is played by cellular contractility mediated by the acto-myosin cytoskeleton, whose action can exert tensile forces on neighbouring cells when those cells are connected together through cell–cell adhesion. We discuss the role that cell–cell adherens junctions (AJ) play in mechanotransduction; describe how epithelia eliminate apoptotic cells by the process of apical extrusion; and discuss how mechanical signalling from apoptotic cells to the surrounding non-apoptotic epithelial cells can underlie apical extrusion. Finally, we consider how the stresses generated during the extrusion process may be limited and resolved to restore mechanical homeostasis to the epithelium.

## A central role for adherens junctions in mechanical cell–cell communication

**Fig. 1:**
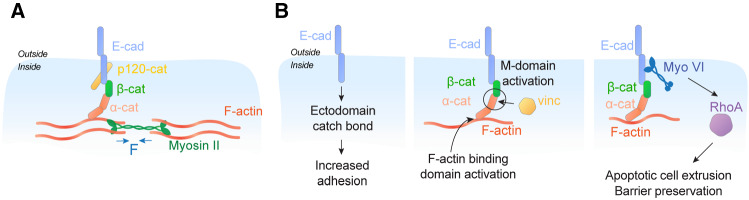
A schematic view of E-cadherin and the mechanical responsiveness of adherens junctions. **A** Schematic view of the core cadherin–catenin complex and its association with the actomyosin cytoskeleton. Note the antiparallel organization of Myosin II motor domains, which is the basis for their generation of contractile force. **B** Mechanosensitive mechanisms in the cadherin apparatus: (left) catch bonds in the cadherin extracellular domain; (centre) mechanosensitive domains in $\alpha$-catenin: the M-domain which recruits proteins such as vinculin, and the F-actin binding domain, which supports a catch bond with actin filaments; (right) an E-cadherin-Myosin VI tension sensor apparatus that activates RhoA signalling (for additional details see [29])

AJ are specialized mediators of cell–cell adhesion, whose core molecular apparatus is the cadherin–catenin adhesion system [10]. This system consists of classical cadherin adhesion receptors (Fig. 1A), membrane-spanning glycoproteins whose extracellular domains mediate cell–cell adhesion and whose cytoplasmic domains recruit $\beta$-, $\alpha$- and p120-catenin in a 1:1:1:1 stiochiometry, as well as a diverse range of other molecules in a sub-stoichiometric fashion. Specifically, $\beta$-catenin binds directly to the cadherin cytoplasmic tail and, in turn, binds to $\alpha$-catenin. $\alpha$-catenin itself can bind to F-actin. Thus, the combination of $\beta$- and $\alpha$-catenin physically couple their cadherin partners to actin filaments (Fig. 1A), through a series of non-covalent protein–protein interactions. This chain of interactions has the effect of linking cadherin adhesion with the actomyosin cytoskeleton, a complex polymeric network built out of F-actin scaffolds and non-muscle Myosin II (NMII) motors [11]. NMII incorporates into the cortex of AJ by binding to actin filaments [12, 13], as it does elsewhere in the cell cortex. The motor activity of NMII allows it to slide actin filaments and NMII also polymerizes to form antiparallel multimers. This combination creates a geometry that generates contractile forces within the actomyosin network when NMII multimers slide F-actin (Fig. 1A and reviewed in detail in [11]). Actomyosin is the major generator of contractile forces in eukaryotic cells and its association with the cadherin adhesion system generates contractile tension at AJ [14].

It is important to note that the cadherin molecular apparatus interacts with a cortical cytoskeleton that is dynamic and subject to strict cellular regulation [15]. First, actin filaments are intrinsically dynamic, being subject to continuous processes of assembly and disassembly [16, 17]. As well, the supramolecular organization of actin filaments is diverse and tightly regulated by the cell: individual filaments are to be found in arrays of branched networks or bundles, which can be linked. Actin filament dynamics and organization are tightly regulated by cell signalling pathways (including Rho family GTPases, lipid kinases and protein kinases) [16]. Second, the recruitment and activation of NMII is itself a response to cell signalling, notably by RhoA and intracellular calcium [11]. AJ themselves recruit many of the cell regulatory mechanisms that modulate actin and myosin dynamics. For example, E-cadherin can directly or indirectly recruit actin nucleators, such as the Arp2/3 complex [18, 19] and formins [20]; guanine nucleotide exchange factors that activate RhoA and Rac, such as Ect 2 [21] and ELMO-DOCK [22], respectively; as well as molecules that control actin organization and turnover, such as Coronin [23] and $\alpha$-actinin-4 [19]. One consequence is that these molecular ensembles can influence the level of contractile tension generated at AJ.

As well as being sites of mechanical tension, AJ possess a number of tension-sensitive molecular mechanisms (Fig. 1B). These take three forms known to date. First, a number of molecules in the cadherin–catenin complex display catch-slip bond behaviour, where their lifetime of binding increases upon application of tension. Catch bonds are found in the adhesive binding extra-cellular domain of the cadherin itself [24], which are thought to promote adhesive strengthening; and also in the binding of $\alpha$-catenin to F-actin [25, 26], which can stabilize the interaction with the cytoskeleton. Second, components of the cadherin complex can change conformation upon the application of tension, revealing cryptic binding sites that recruit additional proteins. The best understood is $\alpha$-catenin, whose central domain opens under tension to promote binding of another actin interactor, vinculin [27, 28], but its catch bond with F-actin is also gated by a conformational change in the actin-binding domain [25, 26].

Third, application of tension to AJ engages a signalling apparatus that can stimulate actomyosin contractility via RhoA [29]. This apparatus involves the unconventional myosin VI, which can interact directly with E-cadherin [30]. The association between these two molecules is enhanced when tension is applied to epithelia. This leads, in turn, to the recruitment and activation of a molecular complex that increases the activity of RhoA. Myosin VI appears to be the proximate mechanosensor in this apparatus, as its association with F-actin is stabilized in response to tension [31].

## Apoptosis in epithelia

Apoptosis is a form of programmed cell death that is elicited by a diverse range of insults (e.g. UV irradiation, toxins) [8, 9, 32]. It is an active process that involves multiple cellular mechanisms, including permeabilization of the mitochondrial membrane with release of mitochondrial contents into the cell cytoplasm, and the activation of caspase proteases, which cleave a staggering number of protein substrates within the cytoplasm [32]. Apoptosis is a homeostatic process, which ideally allows injured cells to be eliminated from the body without provoking inflammatory reactions [32]. As a consequence of the activation of caspases, apoptotic cells characteristically fragment to form membrane-bound apoptotic bodies that can prevent cellular contents from spilling into the extracellular environment within the body to cause inflammation [8]. However, persistent apoptotic bodies can provoke secondary inflammation and autoimmune responses [4]. Thus, the preservation of tissue homeostasis requires mechanisms to eliminate apoptotic cells before these untoward sequelae can occur.

The homeostatic challenge of apoptosis is increased when it occurs within epithelia. Epithelia are membranous tissues that form the principal internal and external barriers of the metazoan body [33]. They range from the single layers of cells that line the gastrointestinal tract or the lung, to the multi-layered, stratified epithelium of the skin and oral cavity. Despite this diversity, a common feature of epithelia is the presence of specialized cell-to-cell junctions that allow cells to adhere to one another and seal the space between cells to prevent the indiscriminate passage of small molecules, fluids and microorganisms [33]. However, barrier function can be compromised when apoptosis occurs within epithelia, both due to fragmentation of apoptotic cells themselves and degradation of the junctions with their neighbouring cells. Moreover, apoptosis is a commonplace event for epithelia, as they are exposed to injurious stresses from the external environment. Characteristically, apoptosis is sporadic, affecting single cells or small clusters of cells, that are surrounded by otherwise uninjured epithelial cells; but this can be sufficient to compromise barrier function [34, 35]. Healthy multicellular life thus requires mechanisms for epithelia to eliminate apoptotic cells.

## Apical extrusion

**Fig. 2:**
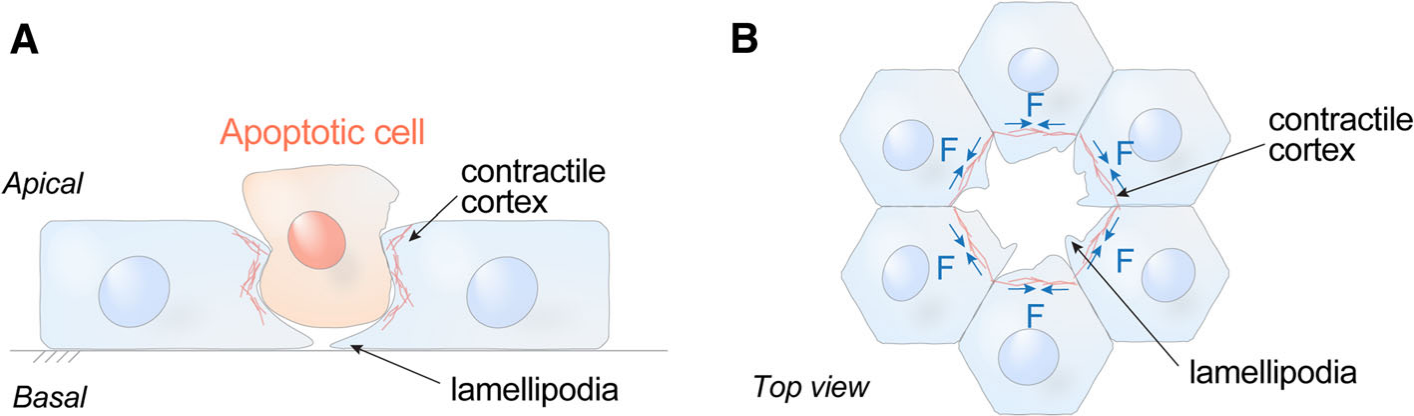
A schematic view of apoptotic extrusion. **A** Side view and **B** top-down view. Sporadic apoptotic cells surrounded by non-apoptotic cells are expelled from the epithelium in an apical direction. The compressive forces responsible for expulsion can be generated by assembly of a contractile cortex in the neighbour cells (located at their interfaces with the apoptotic cell) and/or by the formation of protrusive lamellipodia in the neighbours

Apical extrusion is one response that epithelia use to eliminate apoptotic cells [34]. Extrusion is a morphogenetic phenomenon that occurs in epithelia and endothelia, where small groups of cells are physically expelled in a direction perpendicular to the plane of the epithelium (Fig. 2A). Epithelia are polarized in this axis, with their apical surfaces generally facing the external environment and their basal surfaces contiguous with the body compartment. The extrusion of apoptotic cells commonly (though not always [36]) occurs in an apical direction [34, 37–39], which causes them to be expelled, and thus eliminated, into the external environment (Fig. 2A). Moreover, apical extrusion is a rapid response of the epithelium to apoptosis, typically occurring over a few tens of minutes and often before cells have fragmented [37]. As the apoptotic cell is being expelled, its neighbours also rearrange to ensure that the barrier of the epithelium is maintained [34, 37, 38]. Thus, apical extrusion can prevent inflammatory consequences of epithelial apoptosis from occurring, by simultaneously eliminating apoptotic corpses and preserving the tissue barrier.

An important feature of apoptotic apical extrusion is that it requires the active involvement of the (non-apoptotic) cells located around the apoptotic one [34, 40, 41]. These “neighbour” cells are responsible for generating physical forces to compress and expel the apoptotic cells, as well as altering their own cell shape to preserve the epithelial barrier [38, 41]. Two cellular mechanisms have been implicated in allowing neighbour cells to compress the apoptotic cell (Fig. 2). First, neighbour cells can assemble an actomyosin network at their interface with apoptotic epithelial cells [37, 41, 42], often found at the lateral surfaces of the neighbour cells, extending “downwards” toward their basal pole (Fig. 2A). Second, neighbour cells can generate lamellipodia, veil-like protrusions, oriented toward the apoptotic cell [38, 43]. Lamellipodia are driven by the assembly of branched F-actin networks, whose outward growth extends the protrusions and can compress apoptotic cells.

**Fig. 3:**
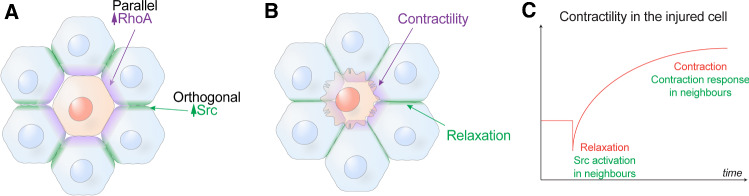
Neighbour cells display disparate mechanical and signalling changes at their cell–cell junctions as they expel apoptotic cells. **A** RhoA is activated in neighbour cells at their interface with the apoptotic cell (parallel junctions) while Src family kinases (Src) are activated at the orthogonal junctions that neighbour cells make with each other. **B** These differences in signals are accompanied by different mechanical changes in the neighbour cell junctions: contractility is increased in the neighbour cells at parallel junctions, but decreased at orthogonal junctions, leading to relaxation of tension at the orthogonal junctions. **C** Schema for the time evolution of mechanical responses in neighbour cells. Initial relaxation of injured cells triggers Src signalling to relax orthogonal junctions; this is succeeded by the onset of hypercontractility as apoptosis proceeds, which activates RhoA in the neighbour cells at their interface with the apoptotic cell

In our discussion, we highlight the role of actomyosin contractility, because it is often necessary for effective apoptotic extrusion, even when lamellipodia are also present [37]. In turn, the assembly of a contractile network reflects the action of cell signalling in neighbour cells (Fig. 3). In particular, the small GTPase RhoA is necessary for contractility and apoptotic extrusion [34]. RhoA is a guanosine nucleotide binding protein which stimulates actomyosin contractility when it is bound to GTP [44]. GTP-RhoA engages a variety of downstream signalling molecules, notably RhoA kinase and formins, which ultimately promote actomyosin contractility. RhoA is activated in neighbour cells [37] and apoptotic extrusion fails when RhoA signalling is blocked in those cells [34], confirming the central role that this contractile regulator plays in the extrusion process. Thus, a key to understanding apoptotic extrusion lies in the process by which an apoptotic epithelial cell signals to activate RhoA in its neighbours.

## Mechanical signalling from apoptotic cells

Cells that are undergoing apoptosis release a wide range of diffusible, chemical signals into their environment [45]. These are commonly implicated in eliciting homeostatic responses from the body, such as the recruitment of innate immune cells to clear apoptotic bodies. Soluble signals have also been proposed to allow apoptotic epithelial cells to signal to their neighbours [46].

More recently, mechanical changes in the apoptotic cells have emerged as a mode for signalling to their neighbours. Specifically, apoptotic cells also display enhanced cellular contractility due to activation of their actomyosin cytoskeleton [47, 48]. NMII can be activated by a number of protein kinases that phosphorylate its regulatory light chain. These contractile kinases (such as Rho kinase and MRCK) are cleaved by caspases during apoptosis [47–49], creating constitutively active forms that drive NMII activation independently of its physiological cellular control. Such hypercontractility is found in many types of cells when they undergo apoptosis and has, indeed, been implicated in apoptotic fragmentation [8].

Several lines of evidence further indicate that apoptotic hypercontractility elicits apical extrusion in epithelia. First, apoptotic epithelial cells display features of hyper-contractility [50] and this is associated with evidence for increased mechanical tension at the junctions between the apoptotic cells and their neighbours [23, 40, 41]. This implied that apoptotic cells could exert mechanical force upon their neighbours. Second, apoptotic hypercontractility is required for apical extrusion, which is blocked when contractility in the apoptotic cell is selectively inhibited [37]. Third, stimulation of contractility within individual cells in an epithelium leads to their extrusion, even in the absence of cell death itself [23, 40]. Together, these findings indicate that an increase in tensile force in the apoptotic cell may be both necessary and sufficient for it to elicit the extrusion response from its neighbours. One possible explanation is that hypercontractility in the apoptotic cell generated a tensile force that was transmitted as a signal to its neighbours. Consistent with this, the activation of RhoA in neighbour cells to generate compression did not occur when contractility in the apoptotic cell was blocked, even though apoptosis continued to proceed [37].

For apoptotic contractility to signal to neighbour cells, it is necessary that there be i) a medium to transmit that mechanical signal between the cells; and ii) cellular mechanisms in the neighbour cells that detect and transduce the tensile signal to stimulate the assembly of a contractile cortex that compresses the apoptotic cell. Because classical cadherin adhesion systems mechanically connect together the contractile cytoskeletons of neighbouring cells, AJ are attractive candidates to transmit increased tension from apoptotic cells to their neighbours. Consistent with this, apoptotic extrusion fails when epithelia are depleted of E-cadherin [40]. Furthermore, as the Myosin VI-based tension sensing apparatus signals to RhoA at junctions, it has the potential to mediate the contractile response within neighbour cells. Indeed, RhoA fails to be activated in neighbour cells when elements of the E-cadherin-Myosin VI mechanotransduction pathway are depleted, and this is accompanied by a failure of apoptotic extrusion itself [37].

Together, these observations yield the following model for the central role of AJ as mechanotransducers in apoptotic extrusion (Fig. 3). While cell–cell adhesion must eventually be lost when apoptotic cells are extruded, this appears to be a late event. Until then, E-cadherin presented on neighbour cells is bound by adhesive *trans-*ligation to E-cadherin on the apoptotic cell. Such *trans-*ligated cadherin mechanically couples the apoptotic cell with its neighbours, allowing enhanced contractility in the apoptotic cell to increase tension on cadherins in the neighbour cell. This stabilizes the association of Myosin VI and E-cadherin to enhance RhoA signalling, leading to the assembly of a contractile cortex in the neighbour cell at its interface with the apoptotic cell. Of note, this enhanced cortex is seen in many, if not all, of the neighbours that abut the apoptotic cell [41]: so, their contraction is predicted to generate a circumferential compressive force to help expel the apoptotic cell.

However, much remains to be learnt about the contractile response of neighbour cells during apoptotic extrusion. Cadherins at steady state can recruit mDia1, a RhoA-activated formin actin nucleator, which contributes to junctional tension [20]. It is therefore tempting to speculate that mDia1 or a similar formin may participate to enhance F-actin in the contractile networks that neighbour cells establish for apoptotic extrusion. This would imply that a significant element of the newly formed F-actin would be nucleated at, and linked to, cadherin adhesion complexes. This is plausible, since E-cadherin adhesive clusters connected to actomyosin are found at the lateral surfaces of cells as well as in the prominent junctions (also known as zonulae adherente) at the apical-lateral interface [51]. But this remains to be tested. Nor should it be assumed that cadherin-associated actin networks are solely linked to cell–cell adhesion. Indeed, actin filaments in neighbour cells have been observed to extend from the interface with apoptotic cells to focal adhesions located at the basal surfaces of the neighbour cells [49]. Finally, we do not yet have high-resolution data on the 3-dimensional organization of the actomyosin networks that neighbour cells assemble to drive apoptotic extrusion. Answering questions such as these will be important to develop a more detailed quantitative understanding of mechanics of apoptotic extrusion.

## Limiting the tissue stress of epithelial apoptosis

In the preceding account, we focused on how contractile forces from apoptotic cells may be transmitted to neighbours at their direct interface with the apoptotic cell (aka “parallel” junctions, Fig. 3A). As well, junctions formed between its immediate neighbours can also intersect with the apoptotic cell. As drawn in Fig. 3A, these junctions are oriented approximately orthogonal to the surface of the apoptotic cell and would be expected to also be affected by tensile forces generated by the apoptotic cell. Indeed, these orthogonal junctions elongate during extrusion, as might be expected if they were being pulled by apoptotic cell. Surprisingly, however, mechanical tension in these junctions was actually reduced when measured, rather than being increased as predicted (Fig. 3B, [52]). Tension in the orthogonal junctions can be considered as the product of pulling forces exerted by the apoptotic cell and also active contractile forces that neighbour cells exert upon one another. Therefore, the observed decrease in tension at the orthogonal junctions could be explained if contractility between orthogonal cells was being reduced. Consistent with this, NMII levels rapidly decreased as the orthogonal junctions elongated [52]. This implies that active relaxation between the neighbour cells may have dominated over the pulling force of the apoptotic cells. This active relaxation process was the product of a different set of cell signals, as it required the action of Src family kinases (SFK) at the orthogonal junctions (Fig. 3A, [52]), which can relax AJ [53, 54]. Moreover, SFK were not activated in response to an increase in tissue tension, but in response to tissue relaxation [52]. Here, closer inspection revealed that one of the earliest observable changes after injury was a transient relaxation of the injured cell, before hyper-contractility became evident, perhaps as an initial response to cell injury before the caspase-dependent apoptotic program was engaged [52]. This suggests a temporal sequence where the injured cell first relaxed, activating SFK in its neighbours, before the apoptotic contractile response became dominant, leading to the RhoA response in the neighbours where they interface with the apoptotic cell (Fig. 3C). Thus, a complex landscape of mechanical changes occurs in neighbour cells during extrusion. While tension is increased at the parallel junctions, it is decreased in the orthogonal AJ.

What function might be served by relaxing orthogonal AJ? One possibility is that this limits the build-up of stress in the monolayer. In addition to the impact of enhanced contractility, implementation of a vertex model predicted that the topological changes associated with loss of apoptotic cells would themselves contribute to mechanical stress in the epithelium if the tissue remains tense during the extrusion process [52]. The model further predicted that apical extrusion would be limited by this build-up of stress, unless mechanisms existed to relax the AJ. Indeed, blocking SFK signalling prevented relaxation of orthogonal junctions and compromised apoptotic extrusion [52]. Thus, apoptotic extrusion requires a closely coordinated pattern of junctional contractility and relaxation: together, these enable the expulsion of the apoptotic cell and allow neighbour cells to rearrange and preserve epithelial barrier function. Similarly, in other morphogenetic contexts, the intercalation of cells between each other requires complementary patterns of active contraction and relaxation at AJ [55, 56].

The relaxation of orthogonal junctions was evident as the apoptotic extrusion process was underway. It may therefore serve as an immediate-early mechanism to limit the build-up of tissue stress that arises during the extrusion process. A longer-term mechanism is to replace the cells lost through extrusion. Proliferation in the neighbours of apoptotic cells can be stimulated by cell-to-cell signalling mediated by the EGF receptor [57], and potentially also in response to increased tissue tension [58]. This would represent a long-term solution to restore tissue topology and would be predicted to operate on a time scale of several hours, depending on the cell cycle duration of the specific tissue involved.

## Future considerations

In summary, we suggest that apoptotic extrusion reflects the mechanosensitive response of neighbouring epithelial cells to apoptotic cells becoming hypercontractile (effectively creating a contractile instability within the epithelium). An implication is that the extrusion process reflects a self-organizing community response to that mechanical instability: exemplifying the notion of a mechanobiome. The cellular mechanisms that eliminate apoptotic cells by extrusion can also be understood to represent tissue responses to a disturbance in mechanical homeostasis, which work— at least in part—to restore mechanical homeostasis. It is likely that the mechanobiome of apoptotic extrusion is guided by other biological and physical parameters, and we highlight some of these to close this review.

We further emphasize that the mechanical changes associated with apoptotic extrusion are likely to reflect feedback interactions between changes in tissue mechanics and cell signalling, mediated by mechanotransduction. For example, change in cortical contractility is likely to be accompanied by changes in cortical stiffness. Cells can detect changes in stiffness at cadherin adhesions [59], so it will be interesting to test if stiffness is another parameter which is detected to influence apoptotic extrusion. Furthermore, other intracellular signals, such as Ca$^{2+\, }$ [60], are activated in neighbour cells during extrusion and likely to affect cell mechanics by regulating the cytoskeleton.

In our discussion, we have focused on a model where apoptosis is predicted to cause a localized disturbance in mechanical homeostasis. But this is not the only paradigm that links tissue mechanics to the morphogenetic process of epithelial extrusion. Nematic analysis of epithelial monolayers has identified local regions of stress which mark sites of apoptotic extrusion [61]. However, in contrast to the system that we have described, in the latter case the nematic defects seem to precede the onset of apoptosis, suggesting that cell death may be a response to alterations in tissue stresses. Tissue overcrowding can also promote the apical extrusion of apparently healthy, live cells [62, 63], which only undergo apoptosis after they have been expelled from the epithelium. Therefore, the morphological phenomenon of apoptotic extrusion may encompass a number of distinct processes, that are distinguished by their dynamic patterns of mechanical change and their accompanying cellular mechanisms.

Finally, it is important to note that apoptotic extrusion is a spatially limited phenomenon. Typically, the cell shape changes and rearrangements of neighbour cells that mediate the extrusion process only occur within a few cell diameters of the apoptotic cell. This suggests that what one might call the “apoptotic mechanobiome” in epithelia is a local phenomenon. The basis for this spatial confinement remains to be identified. Whether it reflects limits of force propagation within the soft materials of tissues and/or active processes that downregulate mechanotransduction or limit force transmission remains to be elucidated. Given the intimate convolution of tissue forces, mechanotransduction and cellular responses that have already emerged, it seems likely to be some combination of these factors.
